# Cigarette Design Features in Low-, Middle-, and High-Income Countries

**DOI:** 10.1155/2012/269576

**Published:** 2012-05-08

**Authors:** Rosalie V. Caruso, Richard J. O'Connor

**Affiliations:** Department Of Health Behavior, Roswell Park Cancer Institute, Elm and Carlton Streets, Buffalo, NY 14263, USA

## Abstract

Previous studies have shown that country income grouping is correlated with cigarette engineering. Cigarettes (*N* = 111 brands) were purchased during 2008–2010 from 11 low-, middle-, and high-income countries to assess physical dimensions and an array of cigarette design features. Mean ventilation varied significantly across low- (7.5%), middle- (15.3%), and high-income (26.2%) countries (*P* ≤ 0.001). Differences across income groups were also seen in cigarette length (*P* = 0.001), length of the tipping paper (*P* = 0.01), filter weight (*P* = 0.017), number of vent rows (*P* = 0.003), per-cigarette tobacco weight (*P* = 0.04), and paper porosity (*P* = 0.008). Stepwise linear regression showed ventilation and tobacco length as major predictors of ISO tar yields in low-income countries (*P* = 0.909, 0.047), while tipping paper (*P* < 0.001), filter length (*P* < 0.001), number of vent rows (*P* = 0.014), and per-cigarette weight (*P* = 0.015) were predictors of tar yields in middle-income countries. Ventilation (*P* < 0.001), number of vent rows (*P* = 0.009), per-cigarette weight (*P* < 0.001), and filter diameter (*P* = 0.004) predicted tar yields in high-income countries. Health officials must be cognizant of cigarette design issues to provide effective regulation of tobacco products.

## 1. Introduction

Tobacco production and consumption have risen dramatically in the developing world [1]. While smoking rates have declined in high-income countries, the public health burden of tobacco is shifting towards the developing world, where by 2030 more than 80% of the world's tobacco-related deaths will occur [2]. Coinciding with this shift to developing countries, health knowledge in these countries is increasing, albeit slowly in some places. While overall awareness of the health hazards of tobacco has improved in the last 15 years in China, it is still relatively poor. A household survey in China found that 81.8% of the population knew that smoking causes serious diseases, but fewer people realized the diseases that second hand smoke could present (64.3%) [3]. Surveys in Ghana, however, show comparatively low smoking prevalence, high awareness of health risks, limited exposure to tobacco advertising, and frequent efforts by smokers to quit [4].

There is evidence that the multinational tobacco industry appears to be targeting Asia and Africa as growth regions [5]. The Framework Convention on Tobacco Control (FCTC), to which 174 countries are currently parties, contains a number of key demand-reducing strategies, such as tobacco taxation, education about health effects (including health warnings on packages), removal of misleading product descriptors, and support for cessation. FCTC also addresses the product itself, and the World Health Organization has received advice from its Study Group on Tobacco Product Regulation on tobacco product testing, reporting requirements, and possible emissions regulation [6, 7]. The problems presented in developing countries will be multifold: to deal with the increasing public health burden, while implementing provisions of the FTC, including educating consumers about the harmful effects of cigarettes and regulating tobacco products.

Over the last five decades, as consumers have grown increasingly aware of the health hazards of smoking, tobacco companies have responded by designing and marketing seemingly lower tar and nicotine products that were positioned as less dangerous to health [8, 9]. However, the testing methodology (e.g., International Organization for Standardization (ISO) and Federal Trade Commission (FTC)) that depicted lower tar and nicotine levels was unrepresentative of human smoking behavior, therefore, labels such as “low tar” often presented on packs or in advertising were meaningless to consumers as health indicators [10]. To market lower tar and nicotine cigarettes, tobacco manufacturers designed their cigarettes with characteristics such as cigarette filters on the ends of rods, which are able to reduce the machine yields of tar and nicotine by 40–50% [11]. Additionally, ventilation holes, which appear as a ring of holes in the cigarette paper surrounding the filter, dilute tobacco smoke coming from the mouth end when smoked by a machine and further reduce tar and nicotine emissions [11]. However, when smoked by consumers, vents can be blocked by fingers and lips, or their effect is reduced by greater puffing effort, such that smokers inhale more tar and nicotine than would be predicted by machine testing [12].

Broadly speaking, cigarette emissions are predictable to a large degree from design features [13–15]. In light of the shifting public health burden of tobacco use toward the developing world, Calafat et al. [16] showed that cigarette emissions and design varied widely across WHO regions, with cigarettes sold in the Eastern Mediterranean, South East Asia, and Western Pacific Regions having higher tar and lower ventilation than those sold in the African, American, or European regions. O'Connor et al. [17] examined the differences in cigarette design characteristics in high-, middle-, and low-income countries, with the general trend being that as country income group increased, cigarettes sold became more highly engineered and the nominal emission levels decreased [17]. All cigarettes from high-income countries had filters, compared with 95% of brands in middle-income and 86% of brands in low-income countries, and among these, the proportion having ventilated filters was 95% in high-income countries, 87.5% in middle income countries, and 44.4% in low-income countries. This current study seeks to replicate earlier findings relating cigarette design (and by extension, emissions) to country development grouping. More evidence from studies such as this one is needed in order for countries to implement meaningful regulation of tobacco, given the important links between cigarette design and smoke emissions [18].

## 2. Methods

Methods for this project mirror a previous study by O'Connor et al. [17], comparing cigarette design features of samples obtained from multiple low-, middle-, and high-income countries. Country income classification was based on the World Bank's Gross National Income per capita data [19]. The current study analyzed cigarettes from 11 countries (*N* = 111 brands) purchased between 2008–2010 (see Table 1). Collaborators in each country purchased popular brands of cigarettes based on sales and prevalence data within each country. Nepal was the only country used in the current study that was also included in the previous study, but these were two separate purchases in two separate years. Packs were then shipped to Roswell Park Cancer Institute where the cigarettes were catalogued and stored at −20°C until analysis. Before testing, cigarettes were conditioned for a minimum of 48 hours at 22 ± 2.0°C and 60 ± 2.0% relative humidity in an environmental chamber.

Product testing procedures followed those previously published by the same laboratory [14, 17]. After conditioning, five cigarettes were selected from each pack for physical analysis. Digital calipers were used to measure the length of the entire cigarette, the length and diameter of the tobacco rod, and the length and diameter of the filter. Filter and tobacco weight measurements were also taken using an analytical balance. The length of the tipping paper was then recorded and observed using a light box for the presence of vent holes. Tobacco moisture and dry weight were assessed using an HR83 Moisture Analyzer (Mettler-Toledo, Columbus, OH, USA). Filter ventilation and pressure drop were assessed using a KC-3 apparatus (Borgwaldt-KC, Richmond, VA). The level of porosity of the cigarette paper was measured using the vacuum method on a PPM1000M paper porosity device (Cerulean, Milton Keynes, UK). Tar and nicotine values were obtained from product packages where these were listed (Table 1).

Data analysis was completed using Statistical Package for the Social Sciences Version 16.0 (SPSS Inc., Chicago, IL, USA). Basic descriptive statistics and analysis of variance (ANOVA) were used to compare product design features by country income grouping. Discriminant function analysis was used to examine how combinations of design features distinguished low-, middle-, and high-income countries. Stepwise linear regression was used to assess the influence of design features on labeled tar and nicotine values. In these regression models, ventilation was forced into the model given extant literature on its major influence on ISO yields [13, 14, 17], while other design features were entered using stepwise procedures (*P*-entry = 0.10, *P*-removal = 0.15). Since tar and nicotine yields were provided on packs for only seven countries (see Table 1), the remaining countries were excluded from the regression analyses.

## 3. Results

Nearly all the cigarettes tested were filtered cigarettes; 100% of cigarettes from both high- and middle-income countries had filters while 89% of cigarettes from low-income countries had filters. Among filtered cigarettes, only 16.0% in low-income countries had vent holes, compared to 65.5% in middle-income countries and 82.1% in high-income countries.

ANOVA analyses (Table 2) revealed basic differences in physical cigarette parameters by income groups in terms of: cigarette length (*P* = 0.001), length of the tipping paper (*P* = 0.010), filter weight (*P* = 0.017), number of vent rows (*P* = 0.003), per-cigarette tobacco weight (*P* = 0.040), ventilation (*P* < 0.001), and paper porosities (*P* = 0.008). The average percentage of cigarette ventilation differed significantly across income groups, with means of 7.49%, 15.34%, and 26.21% for low-, middle-, and high-income groups, respectively, (*P* < 0.001). Rod diameter, filter diameter, tobacco length, and filter length were not shown to have significant differences by income groups.

A discriminant function analysis was used to examine how linear combinations of the panel of design features distinguished among low-, middle-, and high-income countries. Two functions were derived, accounting for 71.4% and 28.6% of variance, respectively. The first function [*X* ^2^ (22) = 45.6, *P* < 0.002] maximally separated the high-income group from low and middle, while the second function [*X* ^2^ (11) = 14.1, *P* = 0.167] separated low- and middle-income groups but did not achieve statistical significance. Examination of the structure matrix suggested that ventilation, paper porosity, cigarette length, and rod diameter distinguished high from the remaining income group brands. Analysis of classification statistics showed that the discriminant functions correctly classified 56.3% of cases, ranging from 72.2% of the high-income brands to 43.5% of the middle-income brands and 69.9% of the low-income brands. 

Stepwise linear regressions were done for all cigarettes with tar and nicotine values recorded on the pack. Per-cigarette weight, tipping paper, filter diameter, tobacco length, and paper porosity were all associated independently with tar yields, after ventilation was forced into the model (Adjusted *R* square = 0.852, see Table 3). For nicotine, ventilation, tipping paper, filter weight, and filter length were the variables predicting nicotine yields (Adjusted *R* square = 0.774; see Table 4). When stratified by income group, regression analyses found that a number of design features contributed independently to tar yields in high-income group countries, including ventilation (*P* < 0.001), tipping paper (*P* = 0.015), number of vent rows (*P* = 0.009), per-cigarette weight (*P* < 0.001), cigarette length (*P* = 0.055), and filter diameter (*P* = 0.004) (Table 3). Middle-income countries had five variables accounting for differences in tar: ventilation, tipping paper length, filter length, number of vent rows in the tipping paper, per-cigarette weight, and cigarette length. In low-income countries ventilation and tobacco length primarily accounted for differences in tar. Ventilation was not statistically significant in both low- and middle-income countries (see Table 3).

When examining correlates of nicotine yield stratified by income group, we found a broadly similar pattern of results (Table 4). In all cases, ventilation and per-cigarette weight had the strongest independent associations with nicotine yield. Other contributors did differ across income groups: filter weight for the low-income (*P* = 0.078), tipping paper length (*P* < 0.001) and filter length (*P* < 0.001) for middle-income countries, and tobacco length for the high-income group countries (*P* = 0.046; see Table 4).

## 4. Discussion

 This study largely replicates an earlier study [17] on the differences in cigarette characteristics between high-, middle-, and low-income countries. As expected, brands in higher income countries were engineered with filters and ventilation more commonly and at higher levels than in lower income countries. Ventilation is the main factor in the differences in tar and nicotine levels among cigarettes [13–15], and a majority of cigarettes in higher income countries employed ventilation to affect tar and nicotine. The main features that distinguished the high-income group brands from the lower income group brands were ventilation, paper porosity, cigarette length, and rod diameter, features which dilute the smoke and/or alter the amount of tobacco available for burning. 

 Patterns in variability in tar across products, by income group, were slightly different than for nicotine. While middle- and low-income countries shared ventilation and tobacco length accounting for most of the variability in tar across their cigarette products, in high-income countries a wider array of design features appeared to have independent influences on tar yields. The added length of the tipping paper is particularly interesting, as it sequesters otherwise smokeable tobacco from burning in a machine test, hence lowering yields [20]. In some countries, maximum tar levels, as measured by standardized smoking machines, have been set, such at the “10-1-10” upper limits for tar, nicotine, and carbon monoxide in the EU [21]. Consumers typically believe products with lower levels to be “healthier”, even though the primary way those numbers are achieved is primarily through increased ventilation. The problem arises in that consumers can directly manipulate how much tar and nicotine they obtain from their cigarettes by blocking the vent holes in the filter or indirectly by taking larger puffs, which ventilation facilitates [11]. In either case, consumers receive more tar and nicotine than stated on the product while believing they have reduced their risks. Given the past history of light and mild cigarettes in developed countries, health officials in developing countries need to be cognizant of these design alterations that can contribute to seemingly “healthier” (i.e., reduced machine-measured tar and nicotine) products introduced into their markets in the coming years. 

Parties to the World Health Organization Framework Convention on Tobacco Control (WHO FCTC) should see this study as further reason to consider cigarette design feature reporting when proposing measures in their countries that regulate the contents and emission of tobacco products (Article 9) and tobacco product disclosures by manufacturers (Article 10) [18]. As noted at COP-4, “Collecting data on product characteristics, such as cigarette design features, would help Parties improve their understanding of the impact these characteristics have on smoke emission levels, properly interpret measurements obtained and, more importantly, keep abreast of any changes to cigarette design features” [18]. In order to have effective product regulation, it is essential that governmental authorities have accurate information about the composition of those products to understand how manufacturers are complying with regulations [18]. 

 A strength of the current study is its consistency with prior findings of statistically significant differences in cigarette design between high-, middle-, and low-income countries, even though completely different sets of cigarettes were tested from different high-, middle-, and low-income countries. The replication of the study further validates the differences in cigarette design between country income groups. At the same time, this study also shared the limitations of the first study [13], that is, the selected brands may not be fully representative of the market within each country. In addition to this, only brands from three low-income countries were tested in this study. Future research on this topic should incorporate more design data from lower income countries. Also, the lower income countries chosen may not be completely representative of all cigarette design from lower income markets around the world. 

 As expected with our hypothesis, the current study shows how different cigarette design characteristics are among high-, middle-, and low-income countries. Smokers in higher income countries have been misled with cigarettes that appear to be less hazardous and have highly engineered cigarette design; lower income countries could avert these same mistakes by immediately establishing ways to regulate product ingredients and design. Public health officials need scientific evidence to better understand cigarette design and function. 

## Figures and Tables

**Table 1:** Summary of countries, income groupings and brands studied.

	Income group	Number of brands	Year pack was collected	Primary manufacturer	T & N label on pack
Bangladesh	Low	5	2009	British American Tobacco	No
Ghana	Low	7	2008	British American Tobacco	Yes
Nepal	Low	16	2009	Other	No
Argentina	Middle	10	2008	Philip Morris	Some packs
Malaysia	Middle	13	2008	British American Tobacco	Yes
Nigeria	Middle	14	2008	British American Tobacco	Yes
Thailand	Middle	10	2008	Thailand tobacco Monopoly	No
Uruguay	Middle	8	2010	Other	No
Canada	High	7	2009	British American Tobacco	Yes
Taiwan	High	11	2008	Taiwan Tobacco and Liquor Corporation	Some packs
UK	High	10	2010	Imperial Tobacco	Yes

**Table 2:** ANOVA, basic differences in physical parameters by income group.

	Income group	Mean	Standard error	Minimum	Maximum	ANOVA	*P*
Cigarette length	Low	79.45	1.24	66.57	84.16	*F*(2,108) = 7.010	0.001
	Middle	82.77	0.34	78.61	93.87		
	High	83.80	1.04	71.79	99.08		
Rod diameter	Low	7.59	0.02	7.34	7.91	*F*(2,108) = 0.079	0.924
	Middle	7.56	0.02	6.84	8.02		
	High	7.54	0.17	2.88	8.02		
Filter diameter	Low	7.58	0.02	7.20	7.77	*F*(2,108) = 0.326	0.723
	Middle	7.60	0.02	6.81	7.85		
	High	7.50	0.18	2.55	7.82		
Tobacco length	Low	61.51	0.56	56.96	68.58	*F*(2,108) = 0.845	0.432
	Middle	60.46	0.49	54.15	70.27		
	High	60.40	0.88	50.21	72.46		
Length of tipping paper	Low	25.70	0.76	18.39	32.65	*F*(2,105) = 4.805	0.010
	Middle	27.98	0.48	15.32	36.40		
	High	28.93	0.87	18.94	38.30		
Filter length	Low	19.92	0.90	8.94	27.23	*F*(2,97) = 2.552	0.083
	Middle	22.89	0.88	11.04	63.26		
	High	21.44	0.71	14.94	26.95		
Filter weight	Low	0.1029	0.0055	0.0458	0.1547	*F*(2,97) = 4.263	0.017
	Middle	0.1178	0.0028	0.0600	0.1556		
	High	0.1172	0.0037	0.0895	0.1585		
Number of vent rows	Low	0.33	0.19	0.00	4.00	*F*(2,93) = 6.226	0.003
	Middle	1.00	0.15	0.00	4.00		
	High	1.46	0.30	0.00	6.00		
Per-cigarette tobacco weight	Low	0.6928	0.0075	0.62	0.77	*F*(2,108) = 3.324	0.040
	Middle	0.6581	0.0116	0.52	1.16		
	High	0.6486	0.0099	0.55	0.75		
Ventilation (%)	Low	7.49	2.3595	0.00	42.22	*F*(2,105) = 2.299	<0.001
	Middle	15.34	1.6746	0.00	39.54		
	High	26.21	3.3641	0.76	68.20		
Paper porosity	Low	35.01	3.16	15.74	80.05	*F*(2,106) = 5.18	0.008
	Middle	44.09	2.40	15.88	81.57		
	High	48.47	2.21	31.42	72.41		

**Table:** (a) Overall

Final adjusted *R*-square value	Model	Standardized coefficients	Sig.
0.852	Vent	−0.722	<0.001
	Per-cig weight	0.475	<0.001
	Tipping	−0.344	<0.001
	Filter diameter	0.233	0.004
	Tobacco length	−0.244	0.017
	Paper porosity	−0.150	0.070

**Table:** (b) Stratified by income group

	Final adjusted *R*-square value	Model	Standardized coefficients	Sig.
Low	0.561	Vent	−0.037	0.909
		Tobacco length	−0.857	0.047

Middle	0.894	Vent	0.055	0.795
		Tipping	−2.139	<0.001
		Filter length	2.212	<0.001
		Number of rows	−0.547	0.014
		Per-cigarette weight	0.620	0.015
		Cigarette length	−0.391	0.072

High	0.956	Vent	−0.897	<0.001
		Tipping	−0.308	0.015
		Number of rows	0.266	0.009
		Per-cigarette weight	0.522	<0.001
		Cigarette length	−0.204	0.055
		Filter diameter	0.282	0.004

**Table:** (a) Overall

Final adjusted *R*-square value	Model	Standardized coefficients	Sig.
0.774	Vent	−0.568	<0.001
	Tipping	−0.752	<0.001
	Filter weight	0.937	<0.001
	Filter length	−0.447	0.059

**Table:** (b) Stratified by income group

	Final adjusted *R*-square value	Model	Standardized coefficients	Sig.
Low	0.860	Vent	−0.191	0.385
		Per-cigarette weight	1.372	0.013
		Filter weight	−0.627	0.078

Middle	0.915	Vent	−0.430	<0.001
		Per-cigarette weight	0.200	0.033
		Tipping	−2.310	<0.001
		Filter length	2.063	<0.001

High	0.710	Vent	−0.637	<0.001
		Per-cigarette weight	0.537	0.003
		Tobacco length	−0.333	0.046
